# Applicability of the linearized Poisson–Boltzmann theory to contact angle problems and application to the carbon dioxide–brine–solid systems

**DOI:** 10.1038/s41598-022-09178-w

**Published:** 2022-04-05

**Authors:** Mumuni Amadu, Adango Miadonye

**Affiliations:** School of Science and Technology, Cape Breton University, Sydney, NS Canada

**Keywords:** Energy science and technology, Engineering

## Abstract

In colloidal science and bioelectrostatics, the linear Poisson Boltzmann equation (LPBE) has been used extensively for the calculation of potential and surface charge density. Its fundamental assumption rests on the premises of low surface potential. In the geological sequestration of carbon dioxide in saline aquifers, very low pH conditions coupled with adsorption induced reduction of surface charge density result in low pH conditions that fit into the LPB theory. In this work, the Gouy–Chapman model of the electrical double layer has been employed in addition to the LPBE theory to develop a contact angle model that is a second-degree polynomial in pH. Our model contains the point of zero charge pH of solid surface. To render the model applicable to heterogeneous surfaces, we have further developed a model for the effective value of the point of zero charge pH. The point of zero charge pH model when integrated into our model enabled us to determine the point of zero charge pH of sandstone, quartz and mica using literature based experimental data. In this regard, a literature based thermodynamic model was used to calculate carbon dioxide solubility and pH of aqueous solution. Values of point of zero charge pH determined in this paper agree with reported ones. The novelty of our work stems from the fact that we have used the LPB theory in the context of interfacial science completely different from the classical approach, where the focus is on interparticle electrostatics involving colloidal stabilization.

## Introduction

Interparticle electrostatics has received the attention of the scientific community owing to its importance in the stabilization of colloids systems^1–3^, in processes related to coal beneficiation^4–6^ and in problems of electrophoretic mobility related to biological or soft matter systems^7,8^. Consequently, the scientific understanding of the nature of the interactions that determine the magnitude and extend of exploitation of such systems for industrial purposes has been sought based on two universally acclaimed theories: the Poisson distribution and the Boltzmann statistical distribution. Poisson’s theory relates to an elliptic partial differential equation, the solution of which gives the potential due to a given electric charge or mass density distribution or ion density, which approximately satisfy the Boltzmann distribution, along with the electrostatic potential. On the other hand, the Boltzmann statistical distribution concerns the probability of finding an ion with a given electrostatic energy^9^. Therefore, the integration of the two theories in physics leads to the Poisson–Boltzmann equation (PBE) in its non-linear form^10^, linearized form (LPB)^11^ and modified form (MPB) which takes into account the finite size of a molecule/ion^12^. Together, they provide a reliable theoretical model for ionic systems and fundamental tools for the industrial applications of such systems.

The PB theory, which shares the assumptions of the mean field theory like the Manning counterion condensation^13^ has been used to gain an insight into ion distribution around charged molecules and ion-mediated interactions between macroions that compare with experimental observations. However, while its success is well documented for systems with monovalent ion concentration^14^ and for low valency ion distributions around charged molecules, the PB theory often fails when the ion charge concentration increases. Manning^13^ suggests that this effect originates from the increase in entropy due to the increase in the effective volume available for condensed counterions as two charged molecules approach each other. The implication is that for very low charged surfaces/molecules, where multivalent electrolytes are involved ion–ion correlations become important due to the large valency of the counterions, and the PB model fails to describe the disjoining pressure acting between charged surfaces even qualitatively^15^.

Furthermore, the shortfall results from the neglect of the interatomic correlation by the classical theory. To address this shortfall, the integral theory, such as the hypernetted chain approximation^16^ and the filed theories^17,18^ have been adopted. The latter has particularly proved used in describing ionic solutions from the weak to intermediate and strong coupling regimes^19^. Generally, the presence of multivalent ions can induce an attractive force between like-charged macroions^20^, the effect of which has been supported by a large number of experiments. A model example is the condensing of DNA molecules by trivalent cobalt-hexamine^21^, multivalent ions mediated network formation in actin solutions^22^. However, for low surface charge density/low surface potential the ion–ion correlation effect is minimal in much the same way as double layer repulsion contribution to disjoining pressure is week under such low surface potential^23^.

Electrostatic/colloidal systems vary in origin. That means differences exist in the potential/charge fields responsible for interactions. Depending on the nature and mechanism of surface charge regulation^24,25^ Bakhshande it is possible to have electrostatic systems with specific ranges of potential, from dilute solutions^26^ with low surface potentials to concentrated solutions with high surface potentials^3^. The former range of potential is the basis for the identification and formulation of the linearized LPB equation.

The scientific validation of the Poisson Boltzmann equation in all three forms has been sought using molecular dynamic simulation, mostly. For instance, the ability of the Poisson–Boltzmann equation to capture molecular dynamics predicted ion distribution around polyelectrolytes has been demonstrated^27^. The numerical solution has also been demonstrated^28^. Moreover, the validity of the LPB equation was demonstrated decades ago using the same technique^29^. Electrostatics interactions found in colloidal systems are like those related to surface complexation in geologic systems, where wetting phenomena are associated with surface charge regulation predictable using the PB theory^30^. In such systems, for instance, those related to geological carbon storage, pH decrease of formation brine leads to decrease surface charge density/potential^31^. Under ambient conditions of saline aquifers, pH is near neutral^32^. For siliciclastic saline aquifers such as sandstones with diagenetic clay minerals^33^ with low point of zero charge pH, the adsorption of counter ions on rock/mineral surfaces results in low surface charge density. Therefore, the addition of hydrogen ions to formation brine during geological sequestration results in further surface charge/potential decrease. The two combined amounts to adsorption and charge neutralization mechanisms^34^. Such a system is, therefore, generally characterized by low surface potential comparable to that based on which the LPB theory is founded. Besides, the Gouy–Chapman model deals with the analytical solution to the PBE which describes the distributions of ions near a planar charged surface in a symmetric electrolyte solution. Moreover, the solution links surface charge density to potential^35^. Therefore, we hypothesize that the analytical development of a quantitative wettability parameter such as contact angle, exploiting such a charge density-potential model provides the basis for testing the validity of the fundamental assumption (low surface potential) enshrined in the Linearized Poisson–Boltzmann equation. Our hypothesis is motivated by the research work of Horiuchi et al.^36^ in which surface potential and surface charge density were calculated by measuring the three-phase contact angle. Accordingly, we have reviewed the basic concepts of surface charge development pertinent to the carbon dioxide–brine–solid system. Following that, we have developed a contact angle equation using the Gouy–Chapman model as an input in addition to a fundamental equation that links contact angle to interfacial free energies under conditions of pH/adsorption induced solid–liquid interfacial energy change^37^. We arrived at a second-degree polynomial based on series expansion of a hyperbolic function, taking into consideration the requirements for such mathematical approaches.

For monovalent ions and weakly charged macroions such as colloids, the classical PB theory makes accurate predictions for ion concentration and distribution as well as for forces between charged surfaces, as predicted by Poisson and Boltzmann theory^38^. In geological sequestration, carbon dioxide will be injected into deep saline aquifers with hypersalinity dominated by sodium chloride ions^39^. Consequently, most recent contact angle measurement on rock minerals to simulate supercritical carbon dioxide dewetting of saline aquifers have been carried out using sodium chloride brine^40–42^. In this regard, the weakness of the classical PB theory is nonexistent because of the predominantly monovalent nature of ions encountered in such systems. In the paper, we apply the linearized PB theory to contact angles measured in such systems for the carbon dioxide–brine–solid systems. Based on differential calculus approach, we have used our contact angle versus pH model to characterize the surface of mica and sandstone samples interacting with supercritical carbon dioxide dissolved species under geological carbon storage conditions, where low surface potentials are imminent due to adsorption of hydrogen ions from dissolved carbon dioxide species in the aqueous phase. We have also discussed the implication of our contact angle model for contact angle discrepancies on Mineral and Rock Surfaces. The novelty of our work stems from the fact that we have used the solution of the PB equation for developing a mathematical tool subject to the low surface potential condition that permits testing the fundamental assumption of the LBP equation.

## Background

### Evolution of the mean-field Poisson–Boltzmann theory

Interfacial forces in aqueous media control electrostatic interactions between charged bodies. In this regard, surface forces ultimately determine whether colloidal particles and macromolecules, such as DNA, proteins, and polymers, will aggregate or will remain stable in dispersions^43,44^. In geologic systems, the electrostatic interactions between fluid–fluid and fluid–solid interfaces contribute to double layer disjoining pressure forces which can be explicitly linked to wetting phenomena^45^. Therefore, accurate mathematical description of the nature of the interactions is critical in diverse scientific disciplines, including those in geologic systems. In the scientific literature, the Poisson–Boltzmann theory provides the fundamental basis for the electrostatic description in such systems in an efficient manner.

However, the basic hypothesis of this robust mathematical tool is that an underlying charged molecular system can be treated as a dielectric medium characterized by its dielectric coefficient that can vary spatially. Under normal conditions, the dielectric coefficient for water is close to 80, while that for a solid such as quartz can be far lower^46^. Moreover, experiment and molecular dynamics (MD) simulations have indicated that the dielectric coefficient can depend on the local ionic concentrations^47^. For instance, the following are the fundamental theoretical basis of PB model:

1. The dielectric permittivity of the solvent is constant and uniform
2. Considers ions to be point sized, and neglects any form of ion–ion correlations
3. In particular, the mean-field PB theory always predicts a repulsive EDL pressure contribution for like-charged surfaces, irrespective of the magnitude of the surface charge density, the ion concentration, or the valence of the counterions. This is in stark contrast to experimental observations.

However, within the framework of surface force, the prediction of the disjoining pressure by the Derjaguin–Laundrup–VeebekOverbeek (DLVO) theory with the PB model as the fundamental basis is in very good agreement with experimental data in the so called weak-coupling effect corresponding to a low surface charge density, high solvent dielectric permittivity, low valence of counterions and high temperature. In the case of multivalent electrolytes, ion–ion correlations become imminent due to the large valence of the counterions. Considering the focus of our paper on the carbon dioxide–brine–solid systems where the ion–ion correlation effect can be encountered, we will devote the following section to a brief but concise review on it and to further evaluate its impact on our results if the need arises.

### The ion–ion correlation effect of Poisson–Boltzmann equation

Considering a multivalent electrolyte, ion–ion correlations become important due to the large valency of the counterions. To integrate the ion–ion correlation effect, Misra et al.^48^ formulated a generalized theory of surface forces, which predicts that the contribution to the disjoining pressure resulting from ion–ion correlations is predominantly attractive, dominating over entropic-induced repulsions for solutions containing multivalent ions. This conceptualization leads to the phenomenon of like-charge attraction. Generally, Ion-specific short-range hydration interactions, as well as surface charge regulation, normally play a significant role at smaller separation distances but do not fundamentally change these trends. Their theory was able to satisfactorily predict the experimentally observed strong cohesive forces reported in cement pastes, which has its origin from strong ion–ion correlations involving the divalent calcium ion.

In addition, to account for the ion–ion correlation effect, Li et al.^49^ considered an ionic solution near a charged surface, assuming there are M ionic species in the solution. (Typically,1 ≤ M ≤ 4.) denote by ci = ci (x) the local ionic concentration of the ith species as a function of distance x. Their key modeling assumption is that the dielectric coefficient ε depends on the sum of local ionic concentrations of all individual ionic (either cationic or anionic). To validate their mathematical analysis, they numerically minimized their electrostatic free-energy functional for a radially symmetric charged system. Their robust computations revealed several features that are significantly different from a system modeled with a dielectric coefficient independent of ionic concentration. These features include the non-monotonicity of ionic concentrations, the previously predicted one-dimensional ionic depletion model near a charged surface and the enhancement of such depletion due to the increase of surface charges or bulk ionic concentrations.

However, in all cases of ion–ion correlation and stearic effects modifications of the original PB Equation, all models converge to the original one at low surface charge density of low potential^50^. This condition of low surface potential/charge characterizes geologic systems under geological carbon storage where contact angles very close 88° have been reported^51^, where all firms of the PB Equations approach linearization. These are the systems we are considering in the context of wetting/contact angle. Therefore, our mathematical modeling will be carried out to demonstrate the applicability of the linearized concept to our research goal.

### The Poisson Boltzmann equation and its linearized form

If two identical rigid spherical colloid particles are separated by a distance and immersed in an aqueous solution of point ions of uniform dielectric potential, where the colloidal particles have uniform dielectric constant equal to that of the electrolyte, the mean electrostatic potential in the surrounding electric double layer is given approximately by the Poisson–Boltzmann equation as^52^:

1a $$\nabla^{2} \psi \left( {r_{1} ,r_{2} } \right) = \left( {8\pi zen/\varepsilon } \right)\sinh (\left( {ze\psi /k_{B} T} \right)$$

In which $\psi$ is the potential, $r_{1} ,\;r{}_{2}$ are radii of two colloidal spherical particle, 1, 2, [m] $n$ is the number density of ions [m^−3^], $z$ is the charge on an ion, $e$ is the electronic charge, $k_{B}$ is the Boltzmann constant [JK^−1^], $T$ is the absolute temperature and $\pi$ is 2.34.

For the case of an isolated charged planar surface at z = 0, suitable for contact angle related problems Eq. (1) can be written as:

1b $$\frac{d\psi }{{dz}} = \eta \sinh \left( {\beta \psi } \right)$$

In which $x$ is the distance from a planar surface [m], $\eta$ is a constant [Cm^−1^] and $\beta$ [C^−1^] a constant.

In its sublimed form, the PBE integrates the phenomenological Poisson equation with that of the Boltzmann statistical distribution *n.* The analytical solution to the PBE predicts the distribution of electrical potential in colloidal/electrostatic systems and forms the basis for predicting the extent of electrostatic interactions/stability of such systems. Electrostatic interactions in physicochemical systems related to minerals, ceramics and the environment are long-range in nature and the accurate analysis of the interactions requires solving the one-dimensional nonlinear Poisson–Boltzmann equation (PBE). Such solutions determine the potential distribution with distance within the electrical double layer relative to the interacting surfaces. Though explicit relations have been developed for the potential profile under certain circumstances^53^, obtaining analytical solutions for a specific situation, such as two interacting plates becomes possible following linearization of the original equation. Such a linearization requires conditions of weakly charged surfaces^54^. Under such conditions, linearization can be achieved subject to the fact that eψ/kT is very small and so eψ/kT is substantially less than 1. This is sometimes called the Debye–Hückel approximation^55^ and the resulting PBE is called the linear Poisson–Boltzmann equation. Following the theoretical basis of hyperbolic sine function, Eq. (1) can be written as:

2 $$\nabla^{2} \psi \left( {r_{1} ,r_{2} } \right) = \left( {8\pi zen/\varepsilon } \right)(\left( {ze/k_{B} T} \right)\psi$$

For the case of an isolated charged planar surface which is attractive for wettability issues, Eq. (2) becomes:^56^:

3 $$\nabla^{2} \psi \left( z \right) = A\sinh \left( {B\psi \left( z \right)} \right)$$

For low surface potential,where

4 $$\sinh (\left( {ze\psi /k_{B} T} \right) \approx \left( {ze\psi /k_{B} T} \right)$$

Equation 2 takes the form^57^

5 $$\nabla^{2} y = \kappa^{2} y$$

where

$$\begin{array}{*{20}l} {\kappa^{2} = \left( {8\pi z^{2} e^{2} n/\varepsilon } \right)} \hfill \\ {y = e\psi /k_{B} T} \hfill \\ \end{array}$$

Equation (2) is a second order linear equation and it is the mathematical basis of the LPBE.

The linearized version of the Poisson–Boltzmann theory is used to calculate the electrical double-layer interaction free energy between identical spherical colloidal particles and for the case in which charge regulation due to the dissociation of surface groups can be modeled by a linear relationship between the surface charge and the surface potential^58^. In line with its fundamental assumption of low surface potential, which characterizes those of mineral/rock in geologic systems under geological carbon storage, we will apply the concept of the LPBE in the appropriate section to achieve the objective of this paper.

### Low surface potential in colloidal systems

Surface charge regulation is an electrochemical process responsible for the development of surface charge density/potential due to protonation or deprotonation of amphoteric surfaces containing ionizable surface functional groups^59^. The magnitude of the surface charge/potential on such surfaces depends on the surface density of surface ionizable groups^60^, pH of aqueous solution^61^ and the ionic strength of aqueous solutions^62^

The development of surface charge/potential on amphoteric surfaces is sensitive to pH and surface chemistry defined by the point of zero charge pH. Below the point of zero charge pH, surfaces adsorb potential determining ions^63^ such as hydrogen ions leading to positive surface charge and potential and vice versa^64^*.* Two mechanisms can lead to low surface potential development: 1. Adsorption of counter ions from solution under conditions of high salinity, 2. Adsorption of proton from solution under conditions of low pH. For instance, the experimental results of Derkani et al.^65^ showed that an increase in concentration of monovalent salt solution (NaCl) results in negative surface charges for both calcite and dolomite particles, but it becomes less negative and its magnitude reduces toward zero with increasing concentration. Generally, low surface potential in geologic or colloidal systems develop due to two main reasons: low surface charge density of ionizable surface groups, for instance, amorphous silica^66^. Under such specific surface chemistry condition, there is a limit to surface charge development due to limited surface sites. Such systems are generally characterized by low zeta potential measurements at a given pH and temperature conditions. Also, experimental results for polyelectrolyte systems show decrease surface charge/potential at a given pH for increasing salt concentration^67^. Consequently, surface modifications have been used to achieve desired performance^68^. In geologic systems, low surface potential arises due to adsorption complexation reactions at surface sites involving aqueous species in solution. Thus, the dissolution of injected carbon dioxide into saline aquifers results in low surface density/potential which results in dewetting of solid surfaces due to thinning and destabilization of the thin water film^69,70^. Prior to the injection of carbon dioxide into saline aquifers, there is equilibrium adsorption of aqueous species on pore surfaces in a manner that reflects hydrogeochemical parameters^71^. Accordingly, surface complexation/adsorption reactions following gas injection constitute a geochemical and adsorption disequilibrium that warrant surface charge regulation and the resulting electrostatic interactions are those that can be described by the PBE. In the following section, we will review the surface complexation reactions associated with such geochemical interactions.

### Surface complexation and charge development in geologic systems

The development of surface charges on mineral surfaces exposed to aqueous media is the consequence of surface complexation, by which water molecules form chemical bonds with under-coordinated surface ions through chemisorption processes^72,73^. In this regard, the surface charge of an oxide/ionizable surface in an aqueous solution is the result of surface reactions involving protonation, deprotonation, and counter-ion association^74^ and the surface charge present at the solid/electrolyte interface is the algebraic sum of the charge bearing groups at the solid surface^75^. Prior to injection into a predominantly sodium chloride formation brine, one important surface complexation reaction can be written for silica dominated saline aquifer rock surface with dissociable surface hydroxyl functional groups. The reaction is^76^:

6 $$SiO^{ - } + Na^{ + } \left( {aq} \right) + H_{2} O\left( {aq} \right) \to SiO^{ - } Na^{ + } + H_{2} O$$

At a given pH of formation brine, surface ionizable groups of silica will dissociate to a degree that is determined by temperature and salinity, producing predominantly negative surface charge density and potential, assuming pH conditions (near neutral-Kharaka et al.^32^ typical of sine aquifers. As injected and dissolved carbon dioxide dissociate into bicarbonate and hydrogen ions^77^ additional surface complexation based on hydrogen ion adsorption onto silica surface will proceed as:

7 $$SiO^{ - } + H^{ + } \left( {aq} \right) + H_{2} O\left( {aq} \right) \to SiOH + H_{2} O$$

At a given pH of aqueous solution, the degree of ionization of surface Silanol group is given as^78^:

8 $$\alpha = \frac{1}{{\left( {1 + 10^{{\left( {pH - pK} \right)}} 2.7^{{Zy_{s} }} } \right)}}$$

In which, $x$ is the degree of dissociation [−], $z$ is the charge; negative for acidic site and positive for basic site, $pH$ is, negative logarithm to base 10 of hydrogen ion concentration, $pK$ is negative logarithm to base 10 of the dissociation constant of surface functional group, $y_{s}$ is the scaled potential defined as^79^:

9 $$y_{s} = \frac{{e\psi_{0} }}{{k_{B} T}}$$

In which, e is the electronic charge [C], T is absolute temperature and $k_{B}$ is Boltzmann constant [JK^−1^].

The net surface charge density is defined as^80^:

10a $$\sigma = F\left( {\Gamma_{ + } - \Gamma_{ - } } \right)$$

In which the $\sigma$ is the surface charge density [Cm^−2^], $F$ is Faraday’s constant [Cmol^−1^], $\Gamma_{ + }$ is the surface excess of positive ions [mol m^−2^] and $\Gamma_{ - }$ is the surface excess of negative ions [mol m^−2^].

The net value of surface charge is calculated as:

10b $$\sigma = F\left[ {\Gamma_{{SiO^{ - } Na^{ + } }} + \Gamma_{{SiO^{ - } H^{ + } }} - \Gamma_{{SiO^{ - } }} } \right]$$

In which the $\Gamma_{{SiO^{ - } Na^{ + } }}$ is the surface excess of surface species $\equiv SiO^{ - } Na^{ + }$ [mol m^−2^], $\Gamma_{{SiO^{ - } H^{ + } }}$ is the surface excess of surface species $\equiv SiO^{ - } H^{ + }$ [mol m^−2^] and $\Gamma_{{SiO^{ - } }}$ is the surface concentration of negatives charge sites $\equiv \Gamma_{{SiO^{ - } }}$ [mol m^−2^]. and

11a $$\equiv \Gamma_{{SiO^{ - } }} = N*\frac{1}{{\left( {1 + 10^{{\left( {pH - pK} \right)}} 2.7^{{zy_{s} }} } \right)}}$$

In Eq. (11), *N* is the surface concentration of dissociable silanol groups [mol m^−2^].

The surface charge density is related to the degree of ionization as:

11b $$\sigma = F\left[ {\Gamma_{{SiO^{ - } Na^{ + } }} + \Gamma_{{SiO^{ - } H^{ + } }} - N*\frac{1}{{\left( {1 + 10^{{\left( {pH - pK} \right)}} 2.7^{{zy_{s} }} } \right)}}} \right]$$

Considering the dependence of the degree of ionization on pH and temperature, surface charge develops in geologic/colloidal systems at a given salinity can be written as, where S denotes salinity:

12 $$\sigma \left( {pH,T} \right)\left| {_{S} } \right. = F\left[ {\Gamma_{{SiO^{ - } Na^{ + } }} + \Gamma_{{SiO^{ - } H^{ + } }} - N*\frac{1}{{\left( {1 + 10^{{\left( {pH - pK} \right)}} 2.7^{{zy_{s} }} } \right)}}} \right]$$

Equation (12) can be correlated to the Gouy–Chapman model in the sense that it links surface charge density to temperature and surface potential through the scaled potential model of Eq. (9). Therefore, in the next section, considering that such systems will be governed by low surface potential, we will exploit the Gouy–Chapman model^35^ for the development of a contact angle model that we believe will be useful in testing the validity of the LPB assumption.

### Theoretical development

#### Contact angle model

Static contact angle is one global quantitative measure of wettability and the direct relationship between zeta potential and contact angle^81^ is indicative of the intimate relationship between contact angle/wettability on surface charge density. In the literature, the relationship between contact angle and interfacial free energies is given by the Young’s equation, which considers an idea and rigid smooth surface, based on mechanical equilibrium concept. The equation is given as^82^:

13 $$\gamma_{LV} \cos \theta = \gamma_{SV} - \gamma_{SL}$$

In which the $\gamma_{LV}$ interfacial tension [Jm^−2^], $\gamma_{SV}$ is the solid–vapor interfacial tension [Jm^−2^], $\gamma_{SL}$ interfacial tension [Jm^−2^] and $\theta$ is the contact angle [°].

The electrochemical properties of the interface are affected not only by the surface chemistry, but also by the particle shape and size and the solution conditions, such as pH and the ionic strength. By tuning these parameters, which is known as charge regulation in the literature, one may have a precise control of the surface charge and related interfacial properties of the colloidal systems^25^. For instance, the interfacial electrical potential difference has been experimentally demonstrated to decrease the interfacial tension in a way that is consistent with Poisson–Boltzmann theory supported by Frenkel–Verwey–Overbeek^83^. Consequently, in line with Eq. (13), any such electrostatic induced change of the solid–liquid interfacial tension will lead to increase in the cosine of the contact angle.

For a pH induced surface charge regulation, the change in solid–liquid interfacial tension relative to its value at the point of zero change pH^84^ takes the following form^85^:

14 $$\gamma - \gamma_{0} = \left( {\int_{{e_{0} }}^{e} {\sigma^{ \pm } } dE^{{^{ \pm } }} } \right)_{{\mu_{{Na^{ + } Cl^{ - } }} }}$$

In which the $\sigma$ is the surface charge density [Cm^−2^], $\sigma$ is the surface charge density [C m^2^], $dE$ is the change in the electrochemical potential of potential determining ions [V], $\gamma$ is the solid–liquid interfacial free energy/interfacial tension [Jm^2^] and $\gamma_{0}$ is the solid–liquid interfacial free energy/interfacial tension at the point of zero charge pH [Jm^2^].

Equation (14) can be linked to the change in electrostatic free energy change as^84^

15 $$\gamma - \gamma_{0} = \left( {\int_{{e_{0} }}^{e} {\sigma^{ \pm } } dE^{{^{ \pm } }} } \right)_{{\mu_{{Na^{ + } Cl^{ - } }} }} = \Delta F_{SL}^{el} \left( {pH} \right) = \sigma_{0} \psi_{0} - \varepsilon_{r} \varepsilon_{0} \kappa \left\{ {\frac{{2k_{B} T}}{e}} \right\}\left[ {\cosh \left\{ {\frac{{e\psi_{0} }}{{2k_{B} T}}} \right\} - 1} \right]$$

In which the $\psi {}_{0}$ is the surface potential [C], $\varepsilon_{0}$ is permittivity of free space [Fm^−1^], is and $\varepsilon_{r}$ the relative permittivity [Fm^−1^].

Henceforth:

16 $$\gamma_{SV} - \gamma_{LV} \cos \theta - \gamma_{0} = \sigma_{0} \psi_{0} - \varepsilon_{0} \varepsilon \kappa \left\{ {\frac{{2k_{B} T}}{e}} \right\}\left[ {\cosh \left\{ {\frac{{e\psi_{0} }}{{2k_{B} T}}} \right\} - 1} \right]$$

Gouy–Chapman theory describes the relationship between the electrostatic potential at the surface and the charge density at the surface. In this paper, we are motivated by the research work of Zhao et al*.*^86^ that provided a concise description of the Gouy–Chapman theory for salt mixtures that is, one of electrolyte mixtures with monovalent and divalent ions (co-ions and counterions) that is applicable to the saline aquifer acting as a geologic repository for anthropogenic carbon dioxide. The model links surface charge density to surface potential as^35,87^:

17 $$\psi_{0} = \frac{{2k_{B} T}}{ze}\sin^{ - 1} \left( {\frac{\sigma }{{\sqrt {8\varepsilon k_{B} C} }}} \right)$$

Equation (16) can be written for surface charge density as:

18 $$\sigma = \sqrt {8\varepsilon k_{B} TC} \sinh \left( {\frac{{\psi_{o} ze}}{{2k_{B} T}}} \right)$$

Substitution into Eq. (18) gives:

19 $$\gamma_{SV} - \gamma_{LV} \cos \theta - \gamma_{0} = \sigma_{0} \psi_{0} - \varepsilon_{0} \varepsilon \kappa \left\{ {\frac{{2k_{B} T}}{e}} \right\}\left[ {\cosh \left\{ {\frac{{e\psi_{0} }}{{2k_{B} T}}} \right\} - 1} \right]$$

At the point of zero charge pH, measured contact angle obeys the following firm of Young’s equation:

20 $$\gamma_{0} = \gamma_{{SL_{PZC} }} = \gamma_{SV} - \gamma_{LV} \cos \theta_{PZC}$$

Substitution gives:

21 $$\gamma_{LV} \cos \theta = \gamma {}_{LV}\cos \theta_{PZC} - \sqrt {8\varepsilon k_{B} TC} \sinh \left( {\frac{{\psi_{0} ze}}{{2k_{B} T}}} \right)\psi_{0} + \varepsilon_{0} \varepsilon \kappa \left\{ {\frac{{2k_{B} T}}{e}} \right\}\left[ {\cosh \left\{ {\frac{{e\psi_{0} }}{{2k_{B} T}}} \right\} - 1} \right]$$

Considering the LPB theory for low surface potential, Eq. (21) becomes:

22 $$\gamma_{LV} \cos \theta = \gamma {}_{LV}\cos \theta_{PZC} - \sqrt {8\varepsilon k_{B} TC} \left( {\frac{{\psi_{0} ze}}{{2k_{B} T}}} \right)\psi_{0} + \varepsilon_{0} \varepsilon \kappa \left\{ {\frac{{2k_{B} T}}{e}} \right\}\left[ {\cosh \left\{ {\frac{{e\psi_{0} }}{{2k_{B} T}}} \right\} - 1} \right]$$

Using series expansion of hyperbolic functions and noting the condition of low surface potential results in accepting the first two terms involving cosh and that leads to the following surface potential dependent contact angle equation:

23 $$\gamma_{LV} \cos \theta = \gamma {}_{LV}\cos \theta_{PZC} - \sqrt {8\varepsilon k_{B} TC} \left( {\frac{ze}{{2k_{B} T}}} \right)\psi_{0}^{2} + \varepsilon_{0} \varepsilon \kappa \left\{ {\frac{{2k_{B} T}}{e}} \right\}\left[ {\frac{1}{2}\left( {\frac{e}{{2k_{B} T}}} \right)^{2} \psi_{0}^{2} } \right]$$

Surface charge develops through several mechanisms, including adsorption of potential determining ions from solution^63^, and each surface charge is responsible for surface potential change ($dE$) (see Eq. (14) the sign of which depends on the sign of the corresponding surface charge. Moreover, from Eq. (23), deprotonated surface site silanol concentration, which is pH depended is the one responsible for surface site density. The pH dependency is intimately linked to the surface potential. Consequently, in Eq. (22), $\psi {}_{0}$ corresponds to the Nernstian potential. The Nernst equation is given as^88^:

24 $$\psi_{0} = - 2.303\frac{{k_{B} T}}{e}\Delta pH = 2.303\frac{{k_{B} T}}{e}\left( {pH_{pzc} - pH} \right)$$

Substitution gives:

25 $$\gamma_{LV} \cos \theta = \gamma {}_{LV}\cos \theta_{PZC} - \sqrt {8\varepsilon k_{B} TC} \left( {\frac{{z2.3^{2} *0.5k_{B} T}}{e}} \right)\left( {pH_{PZC} - pH} \right)^{2} + \varepsilon_{0} \varepsilon_{r} \kappa \left\{ {\frac{{k_{B} T2.303^{2} }}{e}} \right\}\left[ {\left( {pH - pH_{PZC} } \right)^{2} } \right]$$

From Eq. (25), the pH dependent contact angle considering the Gouy–Chapman solution to the PBE and the condition of low surface potential gives:

26 $$\cos \theta = \cos \theta_{PZC} - \left( {\gamma_{LV} } \right)^{ - 1} \sqrt {8\varepsilon k_{B} TC} \left( {\frac{{0.5*z2.302^{2} k_{B} T}}{e}} \right)\left( {pH_{PZC} - pH} \right)^{2} + \left( {\gamma_{LV} } \right)^{ - 1} \varepsilon_{0} \varepsilon_{r} \kappa \left\{ {\frac{{k_{B} T2.303^{2} }}{e}} \right\}\left[ {\left( {pH - pH_{PZC} } \right)^{2} } \right]$$

From Eq. (26), the pH dependent contact angle considering the Gouy–Chapman solution to the PBE and the condition of low surface potential gives:

27 $$\begin{aligned} \theta & = \cos^{ - } \left[ {\cos \theta_{PZC} + \left( {\zeta pH_{PZCeff}^{2} - \zeta 2H_{PZCeff} pH + \zeta pH^{2} } \right)} \right] \\ \zeta & = \left[ {\begin{array}{*{20}c} { - \left( {\gamma_{LV} } \right)^{ - 1} \sqrt {8\varepsilon k_{B} TC} \left( {\frac{z2.302e}{{2k_{B} T}}} \right) } \\ {+\left( {\gamma_{LV} } \right)^{ - 1} \varepsilon_{0} \varepsilon \kappa \left\{ {\frac{{2k_{B} T}}{e}} \right\}\frac{{2.303^{2} }}{2}} \\ \end{array} } \right] \\ \end{aligned}$$

Therefore, for the applicability of Eq. (27) to heterogeneous surfaces, a model of the effective point of zero charge pH is required.

The basis for the derivation of Eq. (27) is the substitution of a surface potential model based on the Nernstian approach. In this regard, contact angles predicted by our model at different pH will deviate from experimental ones where the ion–ion correlation and the steric effects exist. However, in our case, we are interested in low surface potential/surface charge density where these effects are nonexistent, which makes correction of our model for these effects unnecessary. Equations 3 through 4 of Burkhov et al.^50^ show that substitution of very low surface potential value leads to the original form of the PB equation.

### Effective point of zero charge ph for heterogeneous surfaces

The point of zero charge pH is the pH at which the net surface charge is zero. It is a fundamental surface chemistry property of an amphoteric material. Sverjensky^89^ has shown that the point of zero charge pH depends on the crystal chemistry. Accordingly, he linked the parameter to the ratio of the Pauling electrostatic bond strength to the cation-hydroxyl bond length. Therefore, since the surface of a heterogenous amphoteric surface has multiple ionizable groups with different crystal chemistry, an effective value of the parameter is required to represent the surface. For such a surface with different ionizable surface groups responsible for surface charge development where the interaction between surface groups are negligible, the net surface charge density is given for the case of two surface groups as^90^:

28 $$\sigma_{net} = f_{1} \sigma_{1} + f_{2} \sigma_{2}$$

In which this equation, $\sigma_{net}$ is the net surface charge density [Cm^−2^],$f_{1}$ is the fraction of ionizable group 1 [−] and $f_{2}$ is the fraction of ionizable group 2 [−] and $\sigma_{1}$ and $\sigma_{2}$ are surface charge densities of ionizable groups 1 and 2 respectively.

*fk* is moles of OH groups on surface k/total moles of OH groups.

When pH is near the PZC pH of oxide surface, the Nernstian approximation accurately calculates the surfaced charge in terms of pH and point of zero charge pH. However, when the pH is different from the PZC pH of individual surface component, this approximation does not hold, and the following can be written:

29 $$\sigma_{k} = \lambda_{k} K\left( {pH - pH_{PZCk} } \right)$$

where $\sigma_{k}$ is the surface charge density of an ionizable surface group *k* [Cm^−2^],$K$ [−] is a constant to account for the shortfall, $pH$ is logarithm to base 10 of hydrogen ion concentration and $pH_{PZC}$ is logarithm to base 10 of hydrogen ion concentration and $\lambda_{k}$ is moles of OH groups on surface k/ total moles of OH groups [−].

At the point of zero charge pH, the net surface charge is zero, hence for a surface with two ionizable surface functional groups the following can be written for charge neutrality:

30 $$0 = f_{1} \lambda_{1} K\left( {pH_{PZCmix} - pH_{PZC1} } \right) + f_{2} \lambda_{2} K\left( {pH_{PZCmix} - pH_{PZC2} } \right)$$

where $pH_{PZCmix}$ denote the point of zero charge pH of a heterogeneous of mixed surface and $pH_{PZC1}$,$pH_{PZC2}$ are those for surfaces 1 and 2 respectively.

The solution for the effective point of PZC pH of the mixture gives:

31 $$pH_{mix} = pH_{PZCeff} = \frac{{\left[ {Ky_{1} f_{1} pH_{PZC1} + Kyf_{2} pH_{PZC2} } \right]}}{{\left[ {Ky_{1} f_{1} - Ky_{2} f_{2} } \right]}}$$

In which $pH_{PZCeff}$ is the effective point of zero charge pH.

A generalized form of Eq. (31) for several surface ionizable groups can be written as:

32 $$pH_{mix} = pH_{PZCeff} = \frac{{\sum\nolimits_{i = 1}^{n} {Kf_{i} y_{i} pH_{{PZC_{i} }} } }}{{\left[ {Ky_{1} f_{1} - \sum\nolimits_{i = 2}^{n} {Ky_{i} f_{i} } } \right]}}$$

### Validation methods

#### Theoretical Validation of contact angle model

Equation 26 predicts a quadratic relationship between the cosine of contact and pH at a given temperature and pressure while Eq. (27) predicts that for the contact angle and pH. We will assume a seawater-air system where the liquid imbibes into a capillary tube. Considering the equilibrium height, the contact angle can be calculated^91^. Therefore, seawater surface tension is required to validate our model. In this regard, we used a value of seawater surface tension of 73 mNm^−1^ as found in the table of Nayar et al.^92^ corresponding to 19 °C, which is closer to 294 (21 °C) in Table 1. Our objective is to generate data to fit into Eq. (26) so this approximation does not hinder us. To theoretically validate Eq. (26), the parameters found in the equations are necessary.

**Table 1:** Shows parameters and sources.

Parameter	Value	Reference
Boltzmann constant	1.38 * 10^−23^	^93^
Permittivity of free space (Fm^−1^)	8.85 * 10^−12^	^93^
Temperature	318	………
Electronic charge (C)	1.62 * 10^−19^	^93^
Vapor liquid interfacial tension (mJm^−1^)	0.073	^92^
Concentration (mol/l)	0.5	–
Debye length (m) calculation	4.72 * 10^−15^, 1.31 * 10^−8^	
Dielectric constant	74	^94^ Fig. 1

**Figure 1:**
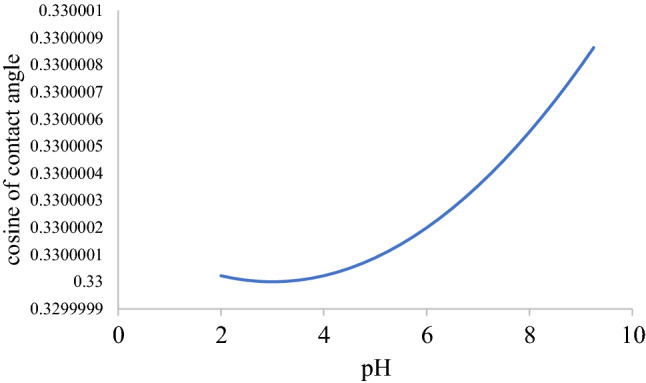
A plot of cosine of contact angle versus pH for 0.5 M sodium chloride brines at 294 K.

Based on the parameters of Table 1, the following figure shows a plot of the cosine of contact angle as a function of pH for 0.5 Molar solution of sodium chloride at 294 K.

Regarding Fig. 1, pH values were assumed. For a set of experimental data involving contact angle and pressure at a given temperature and salinity, the dependence of carbon dioxide solubility in brine on temperature, pressure and salinity^95^ as well as the dependence of the first ionization constant of carbonic acid on these parameters^96^ makes it possible to calculate pH as a function of pressure.

#### pH calculation

In line with the dissolution of carbon dioxide in brine and its subsequent hydration into carbonic acid, the dissociation of the acid will produce hydrogen ions to cause pH evolution with pressure at a given salinity of aqueous solution^97^. Following Miadonye and Amadu^98^, the relationship between pH of aqueous brine containing dissolved carbon dioxide at a given pressure and constant temperature is calculated as:

33 $$pH = \log \left\{ {\gamma \frac{{K_{{CO_{2} }}^{1} \pm \sqrt {\left( {K_{{CO_{2} }}^{1} } \right)^{2} + 4K_{{CO_{2} }}^{1} m_{{CO_{2} }} } }}{ - 2}} \right\}^{ - 1}$$

In this equation, $\gamma$ is the activity coefficient [−], $K_{{CO_{2} }}^{1}$ [M], is the first ionization constant and $m_{{CO_{2} }}$ [M] is the solubility of carbon dioxide in brine [mol L^−1^].

The temperature and salinity dependent ionization constant is given as^96^:

34 $$\begin{aligned} \log K_{{CO_{2} }}^{1} \left( {T,S} \right) & = \frac{ - 2936978}{{T^{2} }} + \frac{17883}{T} - 41.4589 \\ & \quad + \left( {\frac{1141129}{{T^{2} }} + \frac{7220.094}{T} + 13.40776} \right)\sqrt I - 1.414245I + 02677258I^{{{3 \mathord{\left/ {\vphantom {3 2}} \right. \kern-\nulldelimiterspace} 2}}} \\ \end{aligned}$$

In this equation, $\gamma$ is the ionic strength [M].

#### Vapor liquid equilibrium calculation for carbon dioxide solubility

Bahadori et al.^95^ have developed a correlation for predicting the solubility of carbon dioxide in pure water as well as the aqueous sodium chloride solutions based on reduced variables of temperature and pressure and thermodynamic interaction parameters. They applied the solubility model to correlate carbon dioxide solubilities in aqueous solutions for temperatures between 300 and 400 K and pressures from 50 to 700 bars, with good agreement between predicted and experimental data. Their solubility model is given as^95^:

35 $$x = a + bP_{r} + CP_{r}^{2} + dP_{r}^{3}$$

where;

36 $$\begin{aligned} a & = A_{1} + B_{1} T_{r} + C_{1} T_{r}^{2} + D_{1} T_{r}^{3} \\ b & = A_{2} + B_{2} T_{r} + C_{2} T_{r}^{2} + D_{2} T_{r}^{3} \\ c & = A_{3} + B_{3} T_{r} + C_{3} T_{r}^{2} + D_{3} T_{r}^{3} \\ d & = A_{4} + B_{4} T_{r} + C_{4} T_{r}^{2} + D_{3} T_{r}^{3} \\ \end{aligned}$$

### Application of our analytical model

#### Characterization of substrate

##### Cosine of contact angle at zero point of charge

In Eq. (26), temperature and salinity are ambient characteristics of a saline aquifer or geological repository. pH, is a variable, given that carbon dioxide injection, its hydration and subsequent dissociation into hydrogen and bicarbonate ions results in pH changes. Therefore, pH can be varied experimentally. The point of zero charge pH is characteristic of solid surface and must determined experimentally using analytical approaches^99^. The cosine of the contact angle at the point of zero charge pH for a given salinity and temperature also needs to be determined experimentally for a given mineral.

##### Determination of point of zero charge pH from experimental plot

Normally, the solid–liquid interfacial tension increases to a maximum value at the point of zero charge pH, when pH is decreased towards the point of zero charge pH and decreases when pH is decreased from the point of zero charge pH towards lower values^100,101^. Therefore, by Young’s equations, the minimum value of the cosine of the contact angle must occur at the point of zero charge pH. Consequently, given that Eq. (26) is a second-degree polynomial in pH where the cosine of contact angle versus pH describes parabolic curve, the principles of differential calculus can be used to obtain the point of zero charge pH by differentiating it and equating the result to zero. Hence,

37 $$\frac{d\cos \theta }{{dpH}} = \left[ {2\zeta pH_{PZCeff} - 2\zeta pH} \right] = 0$$

Thus:

38 $$pH = pH_{PZCeff}$$

The solution to Eq. (37) shows pH at the turning point is equal to the point of zero charge pH. This approach was used to characterize amphoteric substrates using experimental plots.

### Validation of parabolic model and determination of the point of zero charge pH on amphoteric substrates under geological carbon storage conditions

#### Identification of surface silanol groups mica substrate-literature source information

Due to the availability of experimental data on carbon dioxide-brine-mica system in the literature^42^ we chose this substrate for study. The experimental and technical validity of Eq. (26) stems from pH dependent surface charge regulation, which is characteristic of amphoteric surfaces. Equation (27) calculates contact angle based partly on the effect of solid surface chemistry (point of zero charge pH), which is intimately linked to amphoteric behaviour due to surface functional group protonation or deprotonation reactions and partly on salinity. Therefore, ample evidence of the presence silanol/alunol surface functional groups on mica substrate is essential to validate Eq. (26), as contact angle on substrates has been shown to depend on surface hydroxyl functional groups^59^. This can be done using vibrational spectroscopy^102–104^. Generally, the equilibrium silanol/alunol signal intensity obtained is proportional to the concentration^103^.

**Figure 2:**
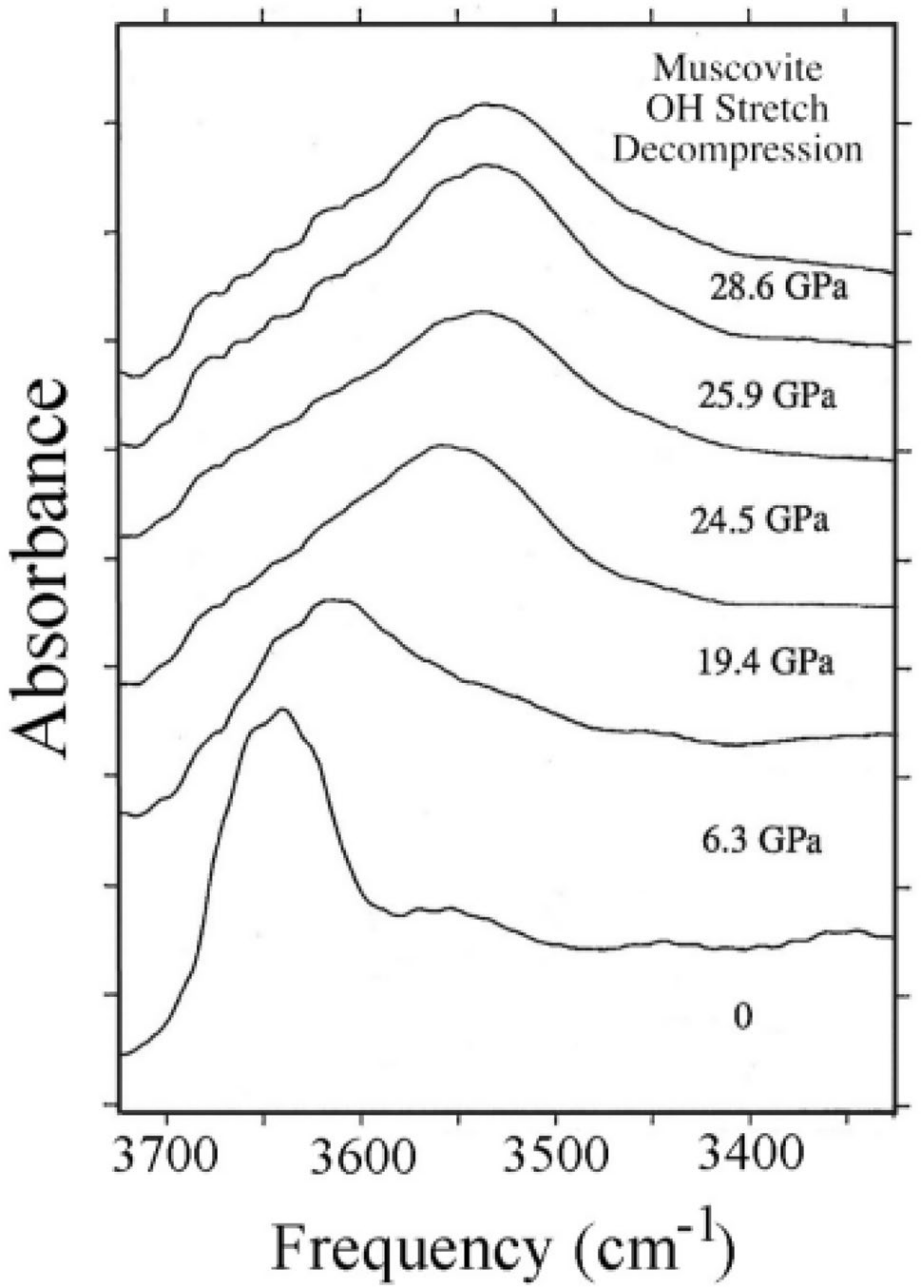
Spectra of the hydroxyl-stretching vibration of muscovite on compression^105^.

Mica is first washed in toluene to remove any organic impurities on the surface. It is then dried in an oven at a temperature of 80 °C overnight. Prepared samples are then kept at room temperature overnight before measurement. Before, acquiring spectra, the substrates must be wiped clean of dust particles that also have the potential to develop surface charges. Spectra for substrates are acquired in the mid-infrared range (400–4000 cm^−1^). Figure 2 shows the infrared spectroscopy of mica under different compressive load from literature source, where the frequency 3650 cm^−1^ corresponds to free silanols.

**Figure 3:**
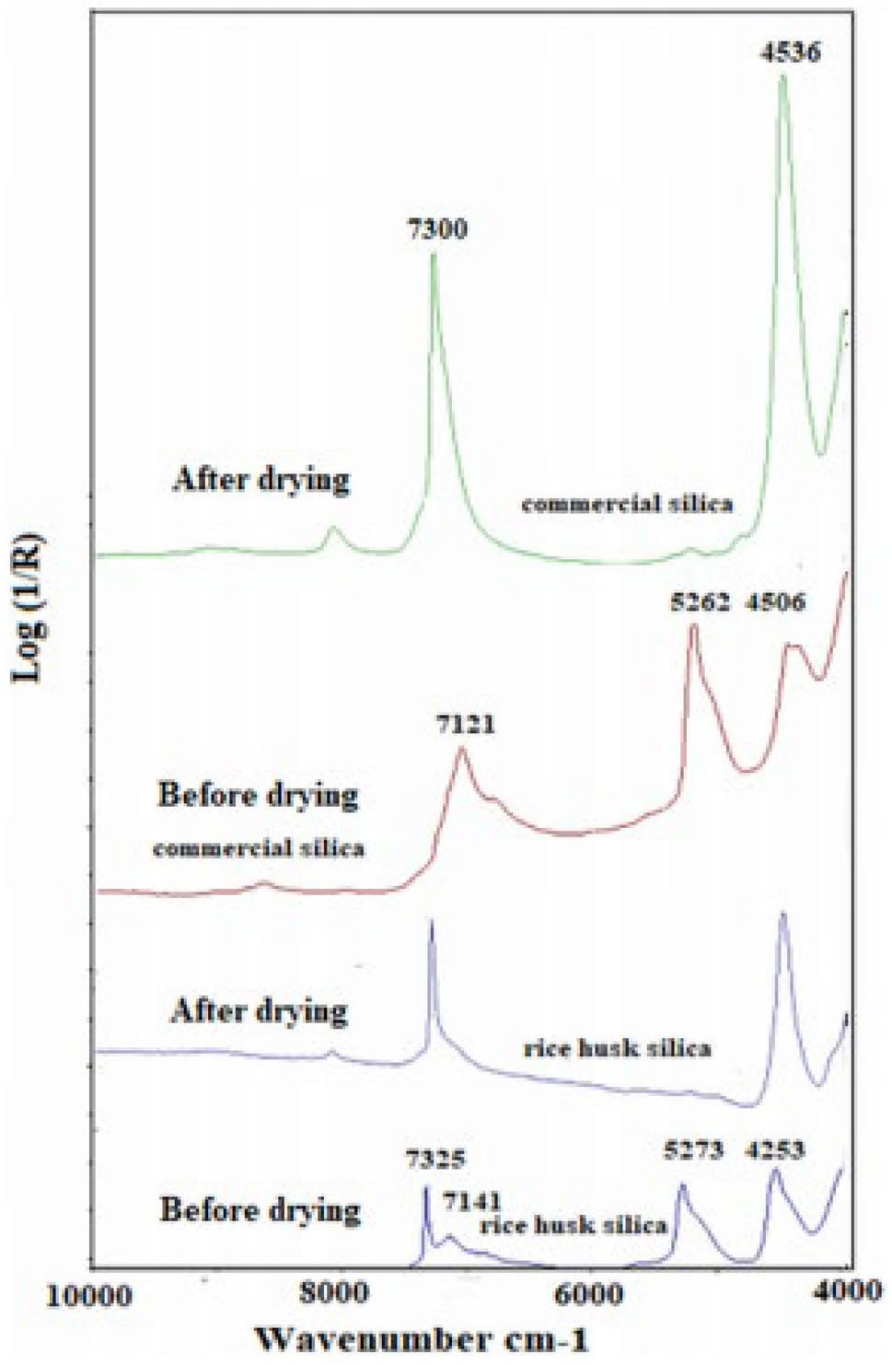
Infrared spectra of quartz^106^.

Similar precautions are followed for acquiring the spectra of silica, but over different frequency range. Figure 3 shows the spectra for quartz with the free silanol OH group occurring at 7316 cm^−1^.

#### Thermodynamic calculations based on mica substrate

**Figure 4:**
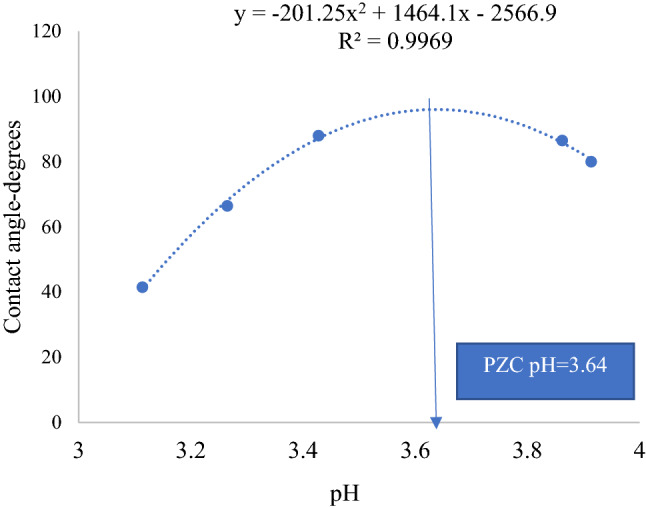
Contact angle versus pH plot for mica substrate.

Our model, Eq. (26), predicts a parabolic plot of cosine of contact angle versus pH, which provides the theoretical justification for the determination of the point of zero charge pH and the contact angle at the point of zero charge pH. When carbon dioxide is injected into saline aquifers under geological sequestration conditions, dewetting of aquifer rock minerals/aquifer rock surfaces will occur in addition to dewetting of a caprock mineral such as mica^107,108^. Jafari and Jung^42^ investigated the variation of contact angle on mica sheet at 318 K, using the captive bubble method for a wide range of pressures and salinities under unsaturated condition. They demonstrated a general increase of contact angle with time. We extracted pressure versus contact angle data from Appendix 1^42^. We calculated solubility versus pressure at the experimental temperature based on Eq. (35) through Eq. (36), using thermodynamic constants from Table 1 of Bahadori et al.^95^. For the one molar solution of sodium chloride we calculated the first-degree ionization constant based on Eq. (34). We calculated pH based on Eq. (33). (The activity coefficient for one molar solution of sodium chloride was taken from^109^ (see Figure 2.14 of Murray^109^) as 0.65. Table 2 shows results of calculations while Fig. 4 shows plot of contact angle versus pH with a regression coefficient of 0.997. The point of zero charge pH is 3.64.

**Table 2:** Calculated solubility and contact angle for mica-brine-carbon dioxide system.

Pressure-MPa	Calculated carbon dioxide solubility-mol/L	Carbonic acid ionization constant using Eq. (34)	Activity coefficient	pH using Eq. (33)	Contact angle from Appendix 1
3	0.009	1.610 * 10^–6^	0.65	3.91	79
5	0.114	1.610 * 10^–6^	0.65	3.86	88
7.8	0.0855	1.610 * 10^–6^	0.65	3.43	87.5
10	0.1812	1.610 * 10^–6^	0.65	3.27	66.5
13	0.3647	1.610 * 10^–6^	0.65	3.11	41.5

#### Thermodynamic calculations based on silica substrate

**Figure 5:**
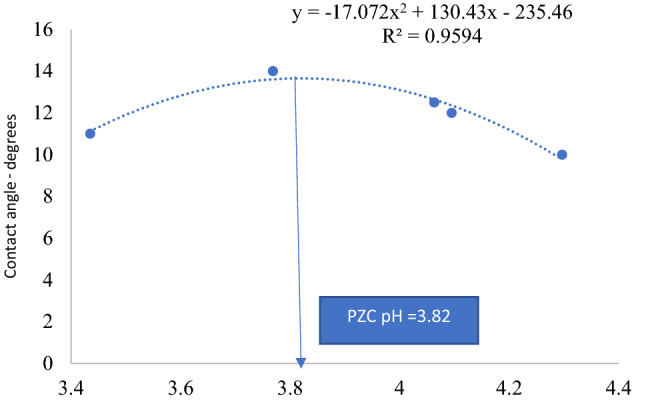
Contact angle versus pH plot for silica substrate.

For the silica–brine–CO_2_ system, we extracted contacted angle versus pressure from the experimental data of Farokhpoor et al.^41^ (see Appendix 2). In this regard, we used the experimental plot for 0 molar sodium chloride solution at 309 K (36 °C). Contact angles were taken to be equal to 180° less the values found in the plot. Solubility calculations were done based on previous approach (see Section 4.2.3). Thermodynamic constants related to 0 molar sodium chloride solution in Bahadori et al.^95^ were used for this purpose. Table 3 shows results of contact angle, calculated solubility and pH while Fig. 5 shows plot for contact angle versus pH with an excellent regression coefficient of 0.997. The point of zero charge pH is 3.82, closer to that reported by Amadu and Miadonye^110^.

**Table 3:** Calculated solubility and contact angle for silica-brine-carbon dioxide system.

Pressure-MPa	Calculated carbon dioxide solubility-mol/L	Carbonic acid ionization constant using Eq. (40)	Activity coefficient	pH using Eq. (39)	Contact angle from Appendix 2
0.5	0.0038	6.488 * 10^–7^	1	4.3	10
2.5	0.0098	6.488 * 10^–7^	1	4.09	12
5	0.0114	6.488 * 10^–7^	1	4.06	12.5
6.5	0.0444	6.488 * 10^–7^	1	3.77	14
10.5	0.2074	6.488 * 10^–7^	1	3.34	11

#### Thermodynamic calculations based on sandstone substrate

**Figure 6:**
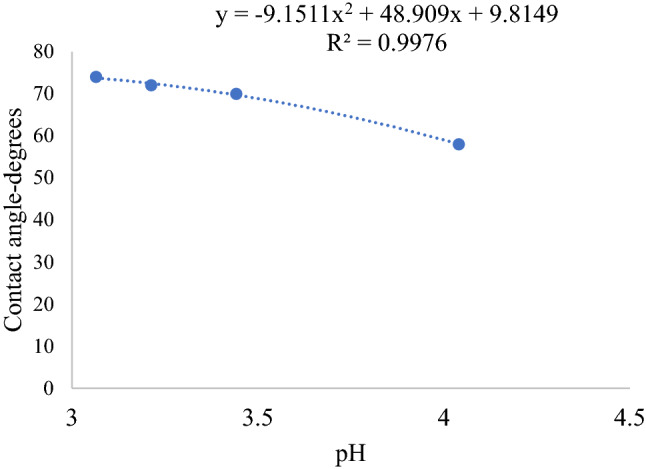
Contact angle versus pH plot for sandstone substrate.

For sandstone calculations, we used advancing contact angles versus pressure measure by Alnili et al.^40^ (see Appendix 3), using deionised water at a temperature of 323 K. Table 4 shows calculated solubilities and pH, while Fig. 6 shows a plot of cosine contact angle versus pressure with the hyperbolic fit having a regression coefficient of 0.998. The point of zero charge pH is 2.67, which is obtained by solving the fitted quadratic equation with regression coefficient close to 1, while the contact angle at the point of zero charge pH is 75°.

**Table 4:** Calculated solubility and contact angle for sandstone-brine-carbon dioxide system.

Pressure-MPa	Calculated carbon dioxide solubility-mol/L	Carbonic acid ionization constant using Eq. (40)	Activity coefficient	pH using Eq. (39)	Contact angle from Appendix 3
5	0.0114	7.17*10^–7^	1	4.04	58
10	0.1812	7.17*10^–7^	1	3.49	70
15	0.5210	7.17*10^–7^	1	3.21	72
25	1.0311	7.17*10^–7^	1	3.06	74

## Discussion

The utility of our model, which is a second-degree polynomial can be seen from its technically feasible validation steps outlined in Section, 4 where important parameters of the aqueous phase, such as the dielectric permittivity and bine salinity can be obtained using models^111^. Also, enough spectral evidence abounds regarding the presence of surface hydroxyl functional groups on rock minerals, such as mica and quartz and, therefore, on rock substrates as seen in Figs. 1 and 2. The dielectric constant can be calculated based on salinity and temperature^111^, while the equivalent sodium chloride concentration concept can be used to calculate the equivalent ionic strength and activity of experimental or formation brine (see Fig. 4 of^112^). Most importantly, the parameters of Eq. (26) that are surface specific regrading charge development that impact contact angle are the point of zero charge pH and the cosine of the contact angle at the point of zero charge pH. These parameters can be deduced from contact angle versus pH data for a given substrate. Based on the validation approaches, experimental data on contact angle versus pH at a given temperature and salinity must describe the polynomial trend represented by Eq. (26).

To stratigraphically trap injected anthropogenic carbon dioxide in saline aquifers until eventual immobilization by mineral carbonation reactions^113^, an efficient cap rock layer on the saline aquifers is essential. These clastic rocks contain phyllosilicate minerals, such as mica. Therefore, their dewetting trends upon contact with supercritical carbon dioxide must be understood to predict the efficiency of stratigraphic trapping in geological carbon storage.

Figure 1 shows a plot of pH versus the cosine of contact angle, using parameters from Table 1. The plot is parabolic with a minimum point at 3, which corresponds to the point of zero charge pH of quartz surface^61^. Accordingly, the plot fits into the contact angle versus pH model in this paper. In our approach, values of pH were assumed to calculate the cosine of contact angle. Therefore, measurement or calculation of pH from experimental data provides the basis for calculating the cosine of contact angle. Consequently, experimental data on contact angle versus pressure for carbon dioxide–solid–brine system should provide the basis for obtaining data on contact angle and pH due to the pressure dependence of carbon dioxide solubility.

Solubility versus pressure data extracted from Jafari and Jung^42^ enabled us to calculated pH versus pressure, which showed a direct correlation of pH to contact angle found in Appendix 1. Table 2 shows results of calculations while Fig. 4 shows the result of fitting a parabolic plot to cosine of contact angle versus pH data based on Eq. (26). The coefficient of regression is 0.997. Accordingly, we deduced the point of zero charge pH of mica used by Jafari and Jung^42^ based on Eq. (38) to be 3.64.

Most phyllosilicate minerals, such as mica have two points of zero charge pH, namely that for the edge plane and that for the basal plane^114^. The protonation and deprotonation reactions of these planes are not the same. For instance, molecular dynamics simulation results have demonstrated that the functionalization of edge surfaces rather than basal planes is more energetically favorable. Consequently, Yan et al.^115^ have determined the point of zero charge pH of mica edge surface to be 7.5. The point of zero zeta potential obtained via high to low pH titration was reported to be located at pH 4.2^116^. Hu et al.^117^ also report that at pH ∼ 3, which was measured for a 50 mM solution, the surface of mica surface is close to its point of zero charge pH. Since Jafari and Jung^42^ measured contacts angle on the smooth basal plane, our calculated value of 3.64, which is 0.56 pH unit lower for one molar sodium chloride solution is theoretically and experimentally meaningful. Moreover, at pH approximately equal to 3, the surface of mica is close to its point of zero charge pH^117,118^. Therefore, the closeness of our calculated point of zero charge pH of mica using in situ experimental data and Eq. (37), which theoretically stems from Eq. (26) gives credence to our pH and salinity dependent contact angle model in this study. Based on the plot (see Fig. 3), the maximum dewetting angle on mica under one molar sodium chloride solution at 318 K, which occurs at the point of zero charge pH is 97°.

Elsewhere^36^, the surface potential and surface charge density at the silica/water surface were calculated by a model based on the Young–Lippmann equation in conjunction with the Gouy–Chapman model for the electric double layer similar to the approach adopted by this paper. In the cited reference, a plot of contact angle versus pH for mica showed that for lower pH values approximate parabolic fit is possible, which deviates for pH values above 4.

For quartz substrate at 309 K and 0 salinity, the point of zero charge pH and cosine of contact angle at this pH are 3.82 and 13.7° respectively based on Fig. 5. Elsewhere^119^, the point of zero charge pH of silica has been reported to range between 2 and 4. The point of zero charge pH and contact angle at this pH deduced from Fig. 6 for sandstone substrate based on our model using experimental data of Alnili et al.^40^ acquired at 323 K with 0 salinity are 2.67 and 75 respectively. Alnili et al.^40^ measured the composition of the sandstone sample using X-ray diffraction (XRD) with a Bruker-AXS D9 Advance Diffractometer and found them to have (quartz 90 wt%, kaolin 3.3 wt%, feldspar 6.7 wt%), and the results indicated that the sandstone sample was composed mainly of quartz. Furthermore, the total organic content is small. Based on our theory (Eq. 31), the point of zero charge pH of sandstone must be higher than that of quartz and this is exactly the case. In the literature, Mushtaq et al.^120^ have obtained a range of point of zero charge pH sandstone with an average of 7.98. However, comparison of the analytical composition of their sandstone (see Table 1 of Mushtaq et al.^120^) to that of Alnili et al.^40^ shows enormous differences in silica or quartz content, such that while the former has 90% quartz, the latter has 65.61% of this mineral. Therefore, the value of 2.67 is meaningful. Moreover, Zafar et al.^121^ have obtained 2.3 for the point of zero charge pH of sandstone for low salinity (see Fig. 3 of Zafar et al.^121^).

Based on our theory (Eq. 31), if quartz is taken to be the reference of a homogeneous component then the sandstone sample of Mushtaq et al.^120^ has more impurities compared to that of Alnili et al.^40^ and would have higher point of zero charge pH, which is consistent with our result. Also, given that contact angle versus pressure for both^40^ sandstone and^41^ substrates were acquired using 0 salinity but different temperatures, the temperature factor can be the major cause for a lower point of zero charge pH of the sandstone. Generally, the point of zero charge pH shifts towards lower values with increase in temperature^122,123^.

Chiquet et al.^124^ have measured contact angles on mica and silica substrates under geological sequestration conditions for salinities ranging from 0. 01 to 1 molar. Their results show that the cosine of contact angle for the silica systems range from 0.82 to 0.93 (see Fig. 7 and Fig. 8 of Chiquet et al.^124^ (see Appendix 4 and Appendix 5), while those for the mica system ranges from 0.4 to 0.94. Therefore, their results confirm values of contact angle at the point of zero charge pH for mica and silica deduced in our study based on our model, where the cosine of the contact angle at the point of zero charge pH is seen to be bigger than that of quartz.

Considering all substates studied in this paper (see Figs. 4, 5, 6), the contact angle decreases as pH decreases or increases away from the point f zero charge pH. This unique trend is explained by increase protonation with pH decrease from the point of zero charge and increase in deprotonation with pH increase from point of zero charge. Both effects can lead to increase surface charge density which can decrease the solid–liquid interfacial tension to reduce contact angle.

Table 5 shows the calculated surface potential versus pH for substrates, using Eq. (24) and the values of point of zero charge pH deduced from plots. On the average, calculated surface potentials are very low, justifying the applicability of the LPB approach in this paper. The table shows that while the surface potentials of quartz and sandstone remained negative throughout, that for mica changed from negative to positive, indicating the development of positive surface charge. The table generally shows decreased surface potential with decreased pH, which is consistent with Fig. 2 of the research work of López-García et al.^125^.The increased positive surface potential of mica after pH of 3.43 shows increased protonation of surface functional groups leading to the development and increase surface positive potential.

**Table 5:** Calculated surface potential versus pH for substrates.

Mica		Quartz		Sandstone	
pH	Surface potential	pH	Surface potential	pH	Surface potential
3.91	− 0.032	4.3	− 0.10	4.04	− 0.088
3.86	0.029	4.09	− 0.087	3.49	− 0.052
3.43	− 0.002	4.06	− 0.085	3.21	− 0.035
3.27	0.008	3.77	− 0.067	3.06	− 0.025
3.11	0.018	3.34	− 0.041		

### Relevance of model to the characterization of rock surfaces

In the literature, the isoelectric point of silica is often determined as the electrokinetic equivalent of the point of zero charge pH. This involves a plot of surface charge density of silica versus pH^126^ where the parameter is determined at zero surface charge density. Therefore, Eq. (26) can be used to further characterize the surface of silica to obtain an integrated approach to characterization, where there is no specific adsorption in which case the point of zero charge pH equals the isoelectric point.

Wettability is a fundamental petrophysical parameters that controls the flow and distribution of fluids phase in porous media^127^. To understand multiphase flow regime involving the flow of injected anthropogenic carbon dioxide and brine in porous media and the extent of dewetting due to the interaction of dissolved aqueous CO_2_ species with aquifer rocks, contact angles have been measured on rock forming minerals^128^**.** For instance, Chique et al.^129^ have measure contact angle on cap rock minerals. Kim et al.^69^ have measured contact angle on silica upon contact with supercritical carbon dioxide and Farokhpoor et al.^41^ measured contact angles on quartz and calcite. Although, measured contact angles on rock minerals reported in the cited literatures reflect water rock interaction of dissolved carbon dioxide species and the minerals concerned under geological conditions of carbon sequestration, they are not representative of the entire aquifer rock domain for some technical reasons^130^. First, rock is defined as aggregate of rock forming minerals^131^. Second, in saline aquifers/depleted oil and gas reservoirs that are the target geologic repositories, digenetic imprints occur as clays and calcite. Consequently, the geological literature distinguishes between quartz arenites^132^ that are predominantly rich in quartz and argillaceous sandstones^133^ having substantial amount of digenetic clays. The division also applies in the case of limestone formations that warrant a distinction between argillaceous limestone^134^ and pure limestone. Therefore, water–rock interaction of dissolved carbon species with actual rock samples will be different from those of unique rock forming minerals (Amadu 2016) and contact angles measured on rock samples under geological conditions of sequestration will be different and completely different from those measured on rock forming minerals.

Considering that rocks are aggregates of rock forming minerals with subordinate or significant amounts of digenetic minerals each with distinct surface chemistry, their isoelectric/points of zero charge pH are equally the aggregate of individual ones. Consequently, the isoelectric point/point of zero charge pH of rock samples have been determined using zeta potential measurement approach^127,135,136^. In recent times, reservoir condition contact angle measurement has become possible, thanks to advances in technology^137–139^**.** Therefore, determination of reservoir condition contact angle on rock samples will provide the opportunity to determine the effective point of zero charge pH of rock surfaces, which reflect in situ surface chemistry of the system. In this regard, the utility of our model (Eq. 26), can be realized from the fact that a plot of reservoir condition contact angle versus pH provides the effective point of zero charge pH at the turning point. Accordingly, Eq. (31) that can be written for the case of an effective point of zero charge pH can be used to determine the sensitivity of reservoir conditions contact angle change to pH change, where aquifer rocks with higher point of zero charge pH values will have lower contact angle sensitivity to pH change. This is because, such higher point of zero charge pH means a narrow range of pH change under actual conditions of geological carbon storage.

### Implications of model for contact angle discrepancies on mineral and rock surfaces

In the geological sequestration literature, contact angles under supercritical conditions have been measured on rock minerals as representatives of actual rocks^41,69^ and on rock samples as aggregates of such minerals^140^. Given the heterogeneity of rock surfaces, the electrokinetic/electrostatic properties of their surfaces, viz point of zero charge pH/isoelectric point will be different from their aggregate minerals. Besides, the composition of impurities has been found to alter the surface chemistry of haematite^141^. Also, discrepancies have been reported on zeta potential measurements on synthetic and artificial calcite^142^. The implication is that the electrostatic properties of surfaces in aqueous systems reflect surface heterogeneity, where surface complexation on minor calcite minerals occurs in a manner comparable to that found in the work of Chen et al.^141^ in addition to those found on major minerals in the rock. Therefore, effective values of the point of zero charge pH or iso-electric points are required to understand the mechanism of surface charge development. Considering our model (Eq. 32), the effective value of the point of zero charge pH is required to understand discrepancies in published experimental data for minerals and rock samples. For instance, the experimental results Kaveh et al.^140^ show that the wettability of Bentheimer sandstone/water system, originates from differences in the surface charges of quartz and Bentheimer sandstone^140^, which must be due to differences in the surface chemistry of the two substrates, which reflect the point of zero charge pH. Zhang et al.^142^ have shown differences in carbon dioxide contact angle for Berea and Obernkirchener sandstones due to differences in rock mineralogy, which affects surface chemistry. They further showed that quartz demonstrated strong water wetness compared to sandstone. In the context of our research work, contact angle discrepancies for rock minerals and rock surfaces can be theoretically explained using Eqs. (27) and (32) together. Thus, from Eq. (27), the bigger the point of zero charge pH, the bigger the contact angle on a substrate. Moreover, the formula for the effective point of zero charge pH for surfaces with multiple ionizable groups (see Eq. 32) that we developed in this paper show that if *i* equals to 1 denotes a homogeneous surface with only one ionizable surface functional group, then any component *i* above 1 denotes an impurity. Accordingly, Eq. (32) shows that the more the impurities on a surface, the lower the denominator and the bigger the effective point of zero charge pH of the surface. The numerator of Eq. (32) also shows that the more the proportion of impurities denoted by *f*_*i*_, the bigger the effective point of zero charge pH. Therefore, considering rock as an aggregate of minerals, the effective point of zero charge pH must be bigger than that those of the aggregate minerals, which correctly explains contact angle discrepancies for rock and mineral substrates as reported in the literature. Normally, as carbon dioxide is injected into saline aquifers, pH change from averagely neutral^32^ to lower pH values. Therefore, based on Eq. (27), the bigger the point of zero charge pH the bigger the contact angle and vice versa*.* Consequently, based on Eq. (27), quartz, which is homogeneous with a smaller point of zero charge pH than sandstone and must show water wetness, which has been experimentally demonstrated^140,142^**.** The dependence of water contact angle on hydroxyl functional groups^59^ of a substrate is further prove that the surface of an aggregate of minerals (rock) must have surface functional group heterogeneity and must have contact angle different from that of a homogenous mineral and this has been theoretically established in this paper.

Equilibrium contact angles plotted versus pH in our work are those described by the Frumkin Derjaguin equation where double layer disjoining pressure component is obtained by solving the PB equation. Where the ion–ion correlation effect is imminent, any mathematical model that uses a surface potential model applicable to the classical theory will not fit well into observations of experimental data. Moreover, in weakly correlating systems such as those specified by low surface potentials, the modified PB equation accounting for the major deficiencies agree with the original equation. The major basis of our work has been the derivation of a quadratic model and the fitting of contact angle versus pH data show very high regression coefficients. Such high regression coefficients of the fits did not warrant any further modification of our model to account for the major defects in the original PB equation by using a modified ion and potential distribution models. Moreover, we studied the carbon dioxide–brine–solid system where pH as low as 3 and closer to the isoelectric point of silica has been reported^143^, where surface charge/potential is low, warranting the adoption of the LPB model to the carbon dioxide-brine-silica and carbon dioxide-brine-mica systems. The reason is that at these low pH conditions closer to their isoelectric points, zeta potentials/surface potentials are very low. For such systems, surface charge regulation under geological carbon storage occurs by adsorption of cations (hydrogen ions from carbon dioxide dissolution) onto negatively charge surfaces. Under such condition, the stearic effect is nonexistent^144^. In addition, for such geological storage systems with high salinities, zeta potentials are generally low with calcite systems being no exceptions^135,136^.

One limitation of the DLVO theory of colloidal stability, and the PB model on which it is based, is the prediction of enhancement of the repulsive disjoining pressure operating between two similarly charged surfaces with increases in surface charge densities (ref-ion–ion correlate). Contrary to this prediction Misra et al.^48^ have demonstrated experimentally that the interaction becomes attractive with increases in surface charge density. The novel theory of the disjoining pressure is due to the existence of ion–ion correlations, which are neglected in the DLVO/PB theory. The implication of the new theory in light of our work is that since increases in surface charge density is the principal cause of the ion–ion correlation effect, double layer repulsion interaction which is linked to contact angle will have no ion correlation effect for low surface potential system like ours. For instance, decreased surface charge/potential of silica under acidic pH of CO_2_ equilibrated water and elevated salinity are predicted to grossly compress the electric double layer, such that the dispersion contribution to film thickness is dominant^70^.

Correcting for the stearic effect due to the finite size of ions that is not accounted for in the PB theory is also not necessary because at low surface potential, the PB theory agrees with its stearic modified one^50^. Moreover, in all the three data sets used for contact angle calculations in our paper, experimental brines are sodium chloride where the multi-valence effect is nonexistent.

## Conclusion

The linear Poisson–Boltzmann equation is built on the premises of low surface potential. Under geological carbon dioxide storage conditions in saline aquifers, the ambient pH of formation brine is near neutral. Consequently, pH decrease with injection will prompt proton adsorption to reduce surface charge density and potential., causing such systems to generally fit into the LPB model. Considering Young’s phenomenological equation for the thermodynamic contact angle, the solid–liquid interfacial energy is central to regulating wettability under variable pH conditions where amphoteric surfaces are concerned. In geologic systems, besides protonation and deprotonation reactions of surface functional groups of rock forming minerals, adsorption of potential determining ions and aqueous species in solution play critical roles in regulating surface charge/interfacial free energy that has been described by the Gouy–Chapman model. Consequently, the surface complexation model is a convenient one to exploit in developing an analytical model that links contact angle to the fundamental parameters of the geologic system, namely pH and salinity. In this research work, we have developed such a model. We have also shown how the model can be theoretically and experimentally validated. We have also demonstrated the applicability of our model based on the LPBE using experimental data on carbon dioxide–brine–solid systems under geological conditions of temperature and salinity taken from the literature. Moreover, we have used our model to theoretically explain discrepancies in contact angle measurement reported for rock surfaces and their aggregate minerals.

The following sum up the conclusion of this study:

1. The fundamental assumption of low surface potential underlying the linear Poisson- Boltzmann theory coupled with the Gouy–Chapman solution of the Poisson–Boltzmann equation leads to the development of a contact angle model that is second-degree polynomial in pH,
2. Considering the low surface potential systems that we studied, we did not have to correct our model for the ion–ion correlation and stearic effects that the original PB theory neglected,
3. Experimental data on contact angle versus calculated pH under conditions of geological storage of carbon dioxide fit into the model with excellent regression coefficients,
4. Our contact angle model contains the effective point of zero charge pH of substrate,
5. Application of the principle of differential calculus enables determination of the point of zero charge pH of a substrate using experimental data of contact angle versus pH,
6. The point of zero charge pH of a micaceous substrate, quartz and sandstone substrates determined using our model compare with literature values,
7. The contact angle/cosine of the contact angle at the point of zero charge pH gives the maximum possible extent of dewetting under pH induced wetting transition of amphoteric surfaces. The model, therefore, provides a tool for experimentally determining the maximum possible dewetting of an amphoteric surface subjected to pH induced surface charge regulation and it can be applied to saline aquifers to obtain an ideal about the extent of carbon dioxide induced dewetting,
8. This paper has, for the first time, demonstrated the applicability of the LPBE using contact angle versus pH data for low surface potential systems characteristic of saline aquifers under geological carbon storage.

## Future research work

While in the Alberta sedimentary basin several potential saline aquifers exist and have been tested for geological carbon storage through research, in Novo Scotia, effort to identify such saline aquifers in the mainland has not been successful to date. Instead, the Carboniferous of Cape Breton is being targeted. Therefore, given the applicability of our model to in situ experimental data pertaining to geological carbon storage that has been convincingly demonstrated in this paper, we intend to acquire in situ experimental data to test the dewetting trends of potential saline aquifers in the future.

## Supplementary Information

Supplementary Information.
