# Supplementary material for: Applicability of the linearized Poisson–Boltzmann theory to contact angle problems and application to the carbon dioxide–brine–solid systems
Source: Sci Rep. 2022 Apr 5;12:5710. doi: 10.1038/s41598-022-09178-w (PMC8983767; doi:10.1038/s41598-022-09178-w)

Appendix 1: Contact angle on mica for CO_2_-brine-mica systems (Jafari and Jung (2018)


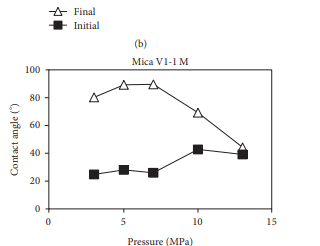


Appendix 2: CO2-brine contact angle on quartz surface versus pressure (Farokhpoor et al., 2013)


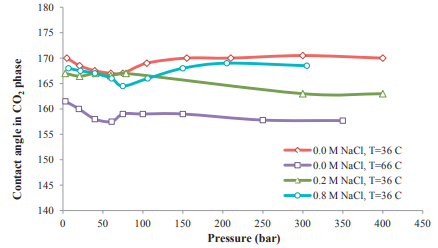


Appendix 3: Receding contact angles for sandstone/CO2/DI water as a function of pressure and temperature; RMS = 540 nm, SD = 3. Alnili, et al., (2018)


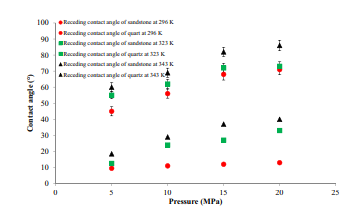


Appendix 4: Figure 7 of Chiquet et al., 2007


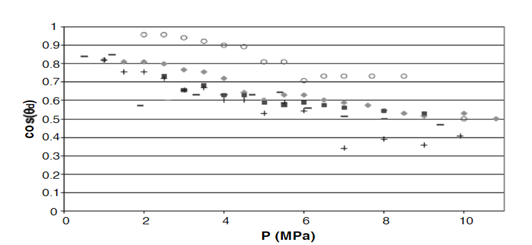


Appendix 5: Figure 8 of Chiquet et al., 2007


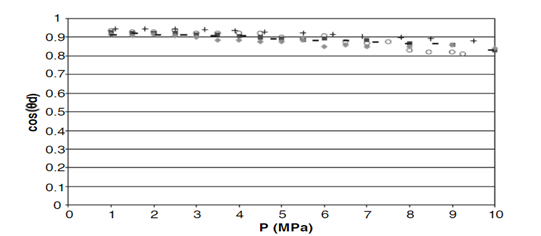

Supplement: Supplementary file 1 — Supplementary Information. [file 41598_2022_9178_MOESM1_ESM.docx]
